# Garnet-Based Solid-State Li Batteries with High-Surface-Area Porous LLZO Membranes

**DOI:** 10.1021/acsami.3c14422

**Published:** 2024-03-04

**Authors:** Huanyu Zhang, Faruk Okur, Bharat Pant, Matthias Klimpel, Sofiia Butenko, Dogan Tarik Karabay, Annapaola Parrilli, Antonia Neels, Ye Cao, Kostiantyn V. Kravchyk, Maksym V. Kovalenko

**Affiliations:** † Laboratory for Thin Films and Photovoltaics, Empa—Swiss Federal Laboratories for Materials Science and Technology, Überlandstrasse 129, CH-8600 Dübendorf, Switzerland; ‡ Laboratory of Inorganic Chemistry, Department of Chemistry and Applied Biosciences, ETH Zürich, Vladimir-Prelog-Weg 1, CH-8093 Zürich, Switzerland; § Department of Materials Science and Engineering, University of Texas at Arlington, Arlington, Texas 76019, United States; ∥ Center for X-ray Analytics, Empa—Swiss Federal Laboratories for Materials Science & Technology, CH-8600 Dübendorf, Switzerland

**Keywords:** LLZO scaffold, membrane, ultrafast sintering, solid-state electrolyte, solid-state Li batteries, Li metal anode

## Abstract

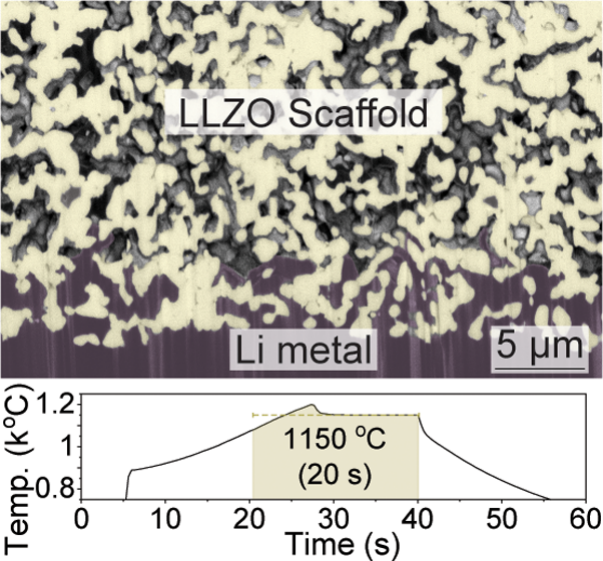

Rechargeable garnet-based solid-state Li batteries hold immense promise as nonflammable, nontoxic, and high energy density energy storage systems, employing Li_7_La_3_Zr_2_O_12_ (LLZO) with a garnet-type structure as the solid-state electrolyte. Despite substantial progress in this field, the advancement and eventual commercialization of garnet-based solid-state Li batteries are impeded by void formation at the LLZO/Li interface at practical current densities and areal capacities beyond 1 mA cm^–2^ and 1 mAh cm^–2^, respectively, resulting in limited cycling stability and the emergence of Li dendrites. Additionally, developing a fabrication approach for thin LLZO electrolytes to achieve high energy density remains paramount. To address these critical challenges, herein, we present a facile methodology for fabricating self-standing, 50 μm thick, porous LLZO membranes with a small pore size of ca. 2.3 μm and an average porosity of 51%, resulting in a specific surface area of 1.3 μm^–1^, the highest reported to date. The use of such LLZO membranes significantly increases the Li/LLZO contact area, effectively mitigating void formation. This methodology combines two key elements: (i) the use of small pore formers of ca. 1.5 μm and (ii) the use of ultrafast sintering, which circumvents ceramics overdensification using rapid heating/cooling rates of ca. 50 °C per second. The fabricated porous LLZO membranes demonstrate exceptional cycling stability in a symmetrical Li/LLZO/Li cell configuration, exceeding 600 h of continuous operation at a current density of 0.1 mA cm^–2^.

## Introduction

The shift from conventional liquid Li-ion electrolytes to nonflammable and nontoxic solid alternatives, harnessing garnet-type structures based on Li_7_La_3_Zr_2_O_12_ (LLZO), has emerged as an effective method for enhancing the energy density, cycling stability, and safety of Li-ion batteries.^1−9^ Despite considerable research efforts, the practical performance of Li-garnet solid-state batteries (SSBs) has not been aligned with commercial requirements. LLZO ceramics, when combined with a Li metal anode, demonstrate reduced cycling stability beyond current densities of 1 mA cm^–2^ and areal capacities exceeding 1 mAh cm^–2^.^10−12^ In such electrochemical scenarios, the rapid penetration of electrodeposited Li into the LLZO solid-state electrolyte (SSE) initiates dendrite formation, leading to potential short-circuiting issues. Moreover, the large-scale production of LLZO SSEs as thin membranes, crucial for achieving high gravimetric and volumetric energy densities, remains a significant practical hurdle.^13−15^

**Figure 1:**
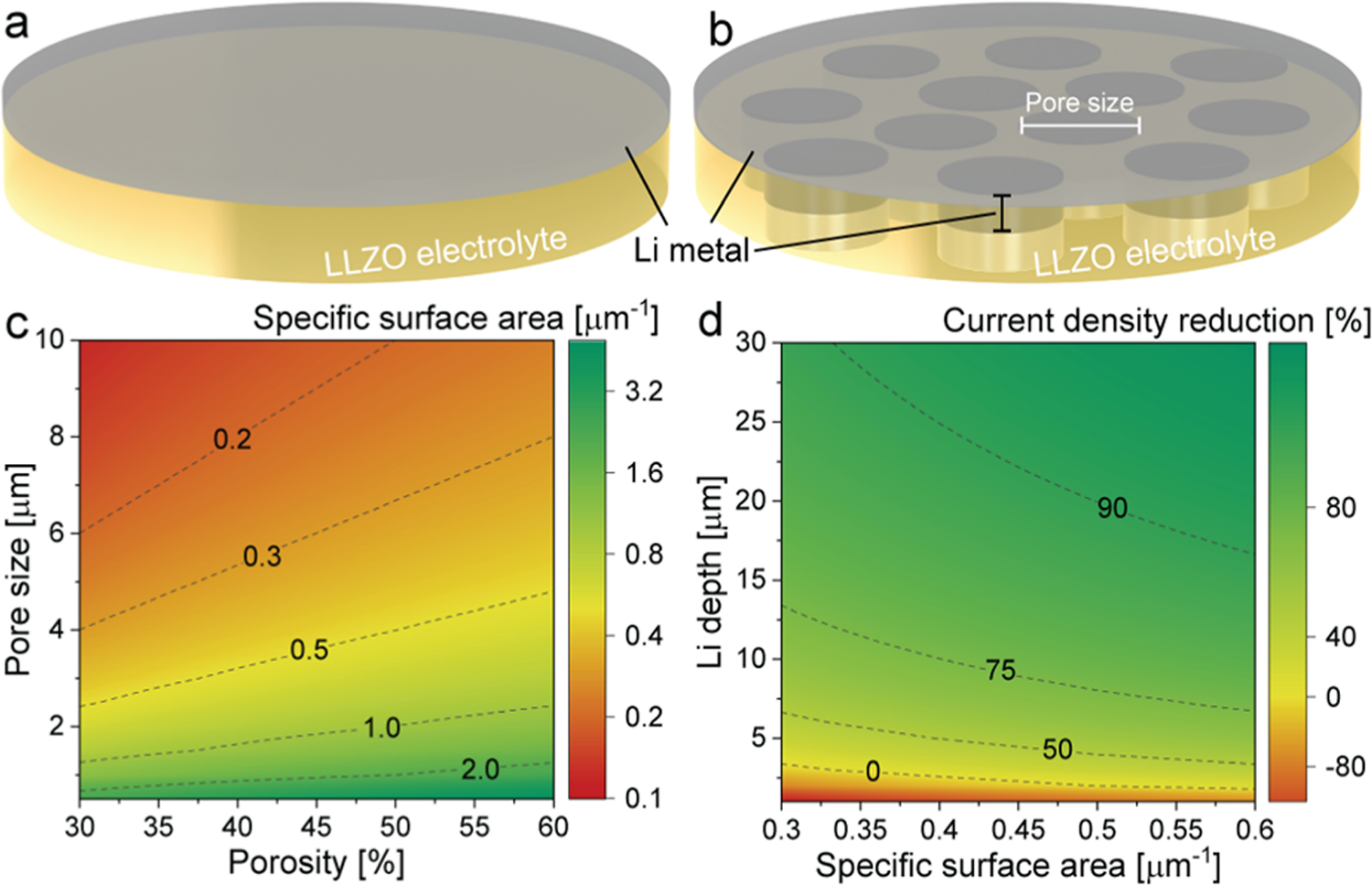
(a, b) Illustration of a flat LLZO surface with metallic Li and a porous LLZO microstructure comprising parallel cylindrical pores, which are filled with metallic Li. (c) Calculated specific surface area of the considered porous microstructure as a function of porosity and pore size. Details of the calculations can be found in the Experimental Section. (d) Calculated reduction in the current density at the contact area between Li and porous LLZO upon Li stripping as a function of the specific surface area and penetration depth of impregnated Li. Note: while experimentally fabricated porous LLZO ceramics exhibit a significantly more complex microporosity than the simplified model of parallel cylindrical pores used in this study, it still allows for estimation of the current density within the porous LLZO.

To address the challenges posed by Li dendrites and the thickness of LLZO solid-state electrolytes (SSEs), recent efforts have focused on the development of thin (30–50 μm) porous LLZO membranes.^16−18^ This porous design aims to tackle the primary factor assumed to cause Li dendrite formation, which is the occurrence of voids during Li stripping from the Li/LLZO interface.^19−25^ Specifically, the approach effectively diminishes the void formation by expanding the interface area between LLZO and Li (Figure 1a,b). Consequently, the amount of Li removed from the interface upon Li stripping is thus drastically reduced, to below that received by the diffusional Li flux toward the Li/LLZO interface, as governed by Fick’s second law. For instance, as illustrated in Figure 1c,d, the use of 1 mAh cm^–2^ Li anodes infiltrated to a depth of 10 μm into a 50%-porous membrane with a pore size of 5 μm leads to a 75% reduction in the applied current density compared with that applied at a flat Li/LLZO interface.

Porous LLZO has been thus far fabricated via diverse approaches.^26−34^ In the initial studies, Fu et al.^35^ introduced a method utilizing poly(methyl methacrylate) (PMMA) pore fillers with a size of 10 μm, resulting in high porosity but relatively large pores of 5–10 μm in the LLZO membranes. Subsequently, Shen et al.^36^ employed *tert*-butyl alcohol crystals as pore fillers in a freeze tape-casting process to fabricate 50 μm porous LLZO structures. Although freeze tape-casting enables the achievement of remarkably high porosities of up to 80%, it has proven ineffective in producing microstructures with small pore sizes below 10 μm. Recently, we have introduced a novel method for fabricating porous LLZO membranes known as intermediate-stage sintering.^34^ This approach involves terminating the sintering process before full densification of the ceramics. A notable advantage of this process is the ability to achieve small pore sizes in LLZO ceramics without the need for pore formers. Yet this pore-former-free approach comes at the expense of the overall porosity and hence reduces the specific surface area. The quest for a suitable method of fabrication of porous LLZO therefore continues since both high porosity and low pore size need to be harnessed in the porous LLZO microstructure.

Motivated by the idea of reducing the pore size of porous LLZO ceramics while maintaining a high level of porosity, in this work, we aimed to develop a fabrication method for porous LLZO membranes that incorporates two pivotal elements into the process: (i) significantly smaller pore formers compared to previous reports and (ii) ultrafast sintering technique (UFS).^37−39^ Owing to the rapid heating/cooling rates during sintering of up to ca. 50 °C per second, UFS prevented substantial ceramics densification, a common issue with the small pore formers. The developed methodology enabled production of membranes with small pore sizes of 2.3 μm and high porosity of 51%, corresponding to a specific surface area of 1.3 μm^–1^, which might well be the highest value for porous LLZO membranes reported to date. To validate the efficiency of the LLZO membranes, we conducted tests in a symmetrical cell configuration. The results demonstrated exceptional cycling stability, surpassing 600 h at a current density of 0.1 mA cm^–2^ with a capacity limit of 0.1 mAh cm^–2^ at room temperature. Importantly, these membranes outperformed their counterparts prepared by using larger pore fillers of 10 μm. The superior performance of LLZO membranes with smaller pores compared with larger sizes was further assessed through phase-field simulations.

## Results and Discussion

### Preparation and Characterization of Porous LLZO Membranes

**Figure 2:**
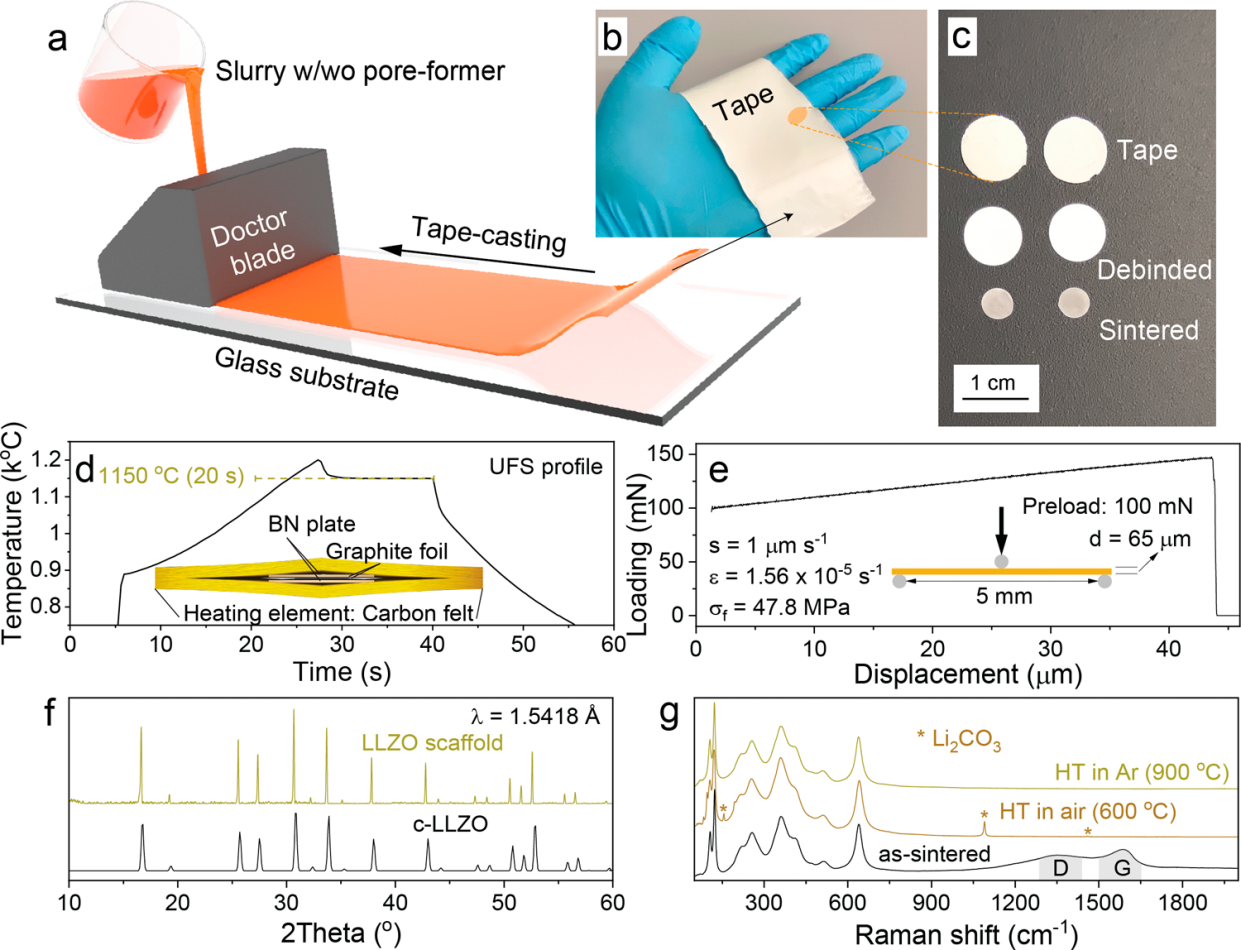
(a) Illustration depicting the tape-casting process of LLZO slurry onto the glass substrate. (b, c) Photographs displaying LLZO membranes at different stages of preparation: post peeling from the glass substrate (b, c), during debinding (c) and subsequent ultrafast sintering (c). (d) A temperature profile utilized during ultrafast sintering of LLZO membranes. (e) A typical force–displacement curve of the LLZO membrane measured by a three-point bending test. (f) X-ray diffraction pattern of the LLZO membrane after heat treatment (HT) at 900 °C in an Ar atmosphere. X-ray diffraction pattern of c-LLZO is given for comparison. (g) Raman spectra of LLZO before and after heat treatment (HT) at 600 °C in air followed by treatment at 900 °C in an Ar atmosphere.

Porous LLZO membranes were fabricated via a process involving tape-casting the LLZO slurry onto a glass substrate, followed by debinding and sintering of the resulting LLZO tapes (Figure 2a–c). The LLZO slurry consisted of LLZO powder, plasticizer, surfactant, binder, solvent (comprising 5 vol % isopropanol, 87 vol % ethanol, and 8 vol % 1-propanol), and 1.5 μm monodisperse acrylic particles serving as pore formers. A detailed description of the fabrication procedure of porous LLZO membranes can be found in the Experimental Section.

Achieving mechanically robust, well-sintered porous ceramics required optimization of the sintering temperature and duration. In this context, considering the very small size of the pore formers, as well as the thickness of LLZO, which could lead to full densification of the LLZO membranes in the case of using relatively low heating and cooling rates during the sintering of membranes in the conventional oven, in this work, we employed UFS for sintering of membranes. The experimental setup for UFS involved two copper electrodes and two superimposed carbon felts clamped between them. The LLZO membrane was placed between carbon foils and BN plates within this setup (Figure 2d).^39^ The selection of BN as a substrate was based on its exceptional thermal stability at operating temperatures, resistance against thermal shocks, and reported high thermal conductivity of ca. 550 W m^–1^ K^–1^.^40,41^ The UFS setup was operated with a direct current power source in an Ar-filled glovebox, and an infrared (IR) camera was used to monitor the temperature of the heating zone.

Sintering experiments on the UFS setup revealed that the optimal sintering temperature and time for the debinded LLZO membrane were 1150 °C and 20 s, respectively (Figure 2d). These conditions resulted in the production of mechanically stable microporous LLZO membranes. Notably, three-point bending tests demonstrated the high mechanical stability of the fabricated LLZO membranes. The breaking force of the 65 μm thick LLZO membrane was measured at 146 mN, corresponding to a flexural strength of 47.8 MPa at 43.7 μm displacement (Figure 2e), which aligns well with findings from previous studies on LLZO membranes.^23,34^ Employing lower sintering temperatures or shorter durations resulted in the production of exceedingly fragile LLZO membranes without proper sintering (Figure S1a,b). Conversely, higher sintering temperatures or extended sintering durations led to overdensified membranes (Figure S1c,d). X-ray diffraction (XRD) analysis of the membranes sintered at 1150 °C for 20 s (Figure 2f) validated the formation of a fully cubic LLZO structure (*Ia*3̅*d*, *a* = 12.9622(2) Å, *V* = 2177.89 Å^3^, ICSD 235 896). Importantly, we find the debinding of the LLZO tapes under O_2_ flow is essential for obtaining an impurity-free cubic LLZO phase, as compared to the debinding in air under stationary conditions without flow (Figure S2). This observation can be explained by the increase in the efficiency of CO_2_ removal, formed as a result of burning organic components during debinding of LLZO membranes. Therefore, the decrease in the concentration of CO_2_ on the LLZO surface mitigates its chemical reaction with LLZO and, therefore, the formation of Li_2_CO_3_ and La_2_Zr_2_O_7_ secondary phases.

To evaluate the chemical purity of the LLZO surface following the UFS process, Raman spectroscopy was employed. Figure 2g displays the Raman spectra of the sintered LLZO membranes, indicating distinguishable G and D bands, characteristic of graphite impurities from the graphite foil employed as the substrate during UFS. To eliminate the graphite contamination, a two-step heat-treatment procedure was performed. The sintered membranes were first subjected to heat treatment in air at 600 °C for 30 min, followed by an additional annealing step in an Ar-filled glovebox at 900 °C for 10 min. Importantly, the Raman peaks at 155 and 1090 cm^–1^, attributed to Li_2_CO_3_, vanished after the 900 °C heat treatment in Ar, confirming the successful elimination of Li_2_CO_3_ impurities from the LLZO surface. This elimination is crucial, as their presence significantly elevates the Li/LLZO interfacial resistance and fosters Li dendrite formation.^42^ Importantly, the Raman peaks within the range 100–600 cm^–1^, representing the characteristic cubic structure of LLZO, remained consistent throughout the heat-treatment process. X-ray photoelectron spectroscopy (XPS) measurements further validated the removal of Li_2_CO_3_ impurities from the LLZO surface (refer to Figure S3 for charge-corrected Zr 3d and O 1s spectra of ultrafast-sintered LLZO membranes after heat treatment in air followed by Ar-filled glovebox heat treatment at 900 °C).

**Figure 3:**
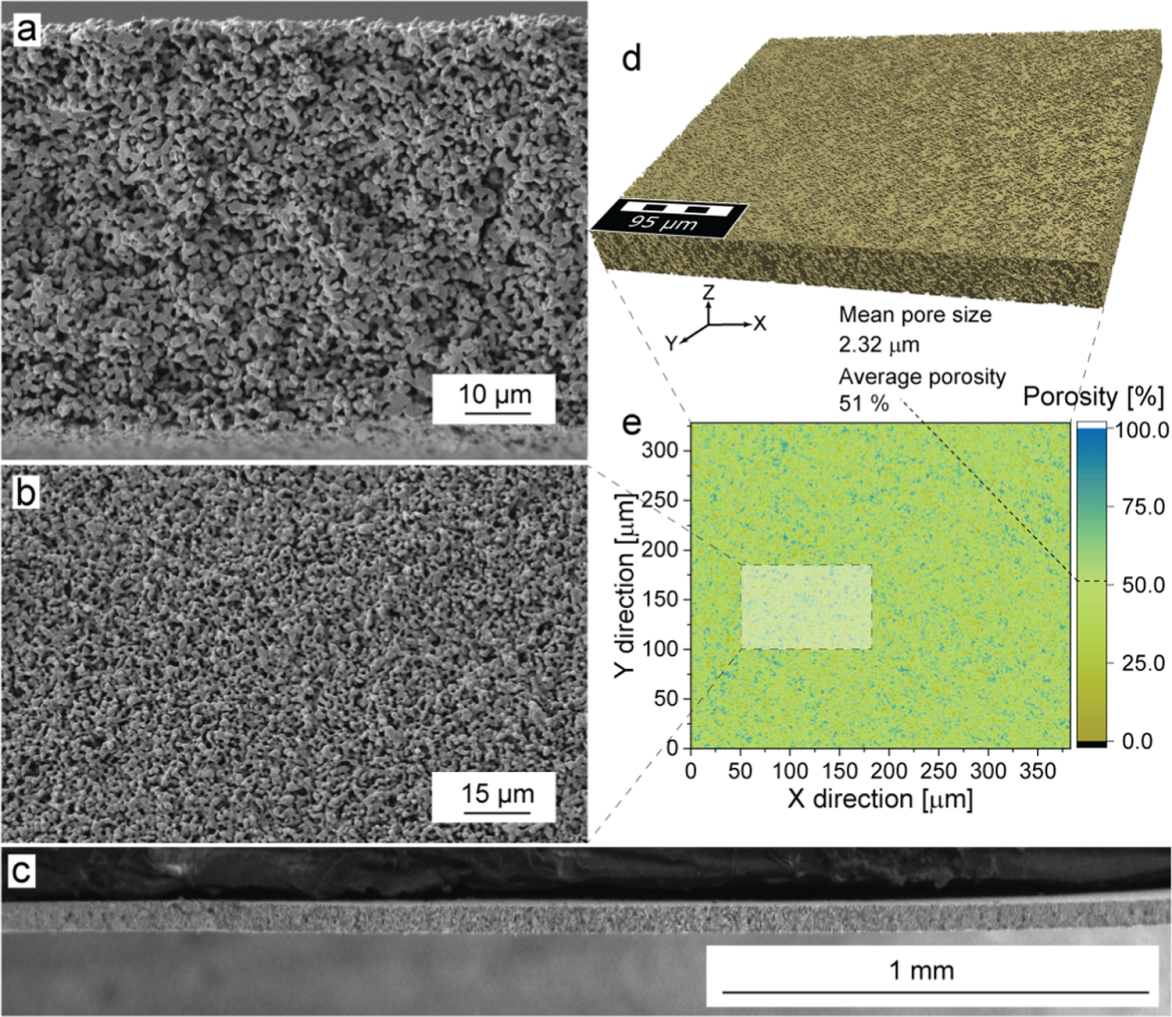
(a–c) Cross-sectional (a, c) and top-view (b) SEM images of sintered LLZO membranes. (d) X-ray microcomputed tomography image of the sintered LLZO membrane. (e) Computed porosity of the LLZO membrane along the *Z*-axis (thickness) derived from XCT analysis. More comprehensive details regarding the analysis of the reconstructed three-dimensional (3D) images of LLZO membranes can be accessed in the Experimental Section.

The analysis of the fabricated membranes using scanning electron microscopy (SEM, Figure 3a–c) and X-ray microcomputed tomography (CT, Figure 3d,e) confirmed the successful fabrication of scaffolds with the average pore size of ca. 2.3 μm and the average porosity of ca. 51%. This combination of a low pore size and high porosity resulted in an exceptionally high specific surface area of 1.3 μm^–1^, likely surpassing all previously reported LLZO scaffolds (see Figure S4 and Table S1). Importantly, the X-ray tomography analysis revealed that the scaffold consisted solely of open-pore channels (Table S2).

To increase the porosity of the LLZO membranes, we experimented with higher volumetric contents of the pore formers in the slurry, exceeding 87.5%. However, these efforts resulted in the formation of inhomogeneous slurries, despite our adjustments to the surface of the pore formers by increasing the concentrations of surfactants and plasticizers. As a consequence, tape-casting these slurries led to the formation of defective LLZO membranes. Additionally, it should be noted that the use of smaller pore formers generally leads to lower porosity in sintered porous ceramics compared to larger ones, even when the same initial volumetric content of pore formers is employed (Figure S5). This phenomenon is commonly observed in various ceramic materials, including LLZO, highlighting the importance of optimizing the selection and size of the pore formers to achieve the desired microstructure in the final ceramic membranes.

### Electrochemical Performance and Phase-Field Simulations

**Figure 4:**
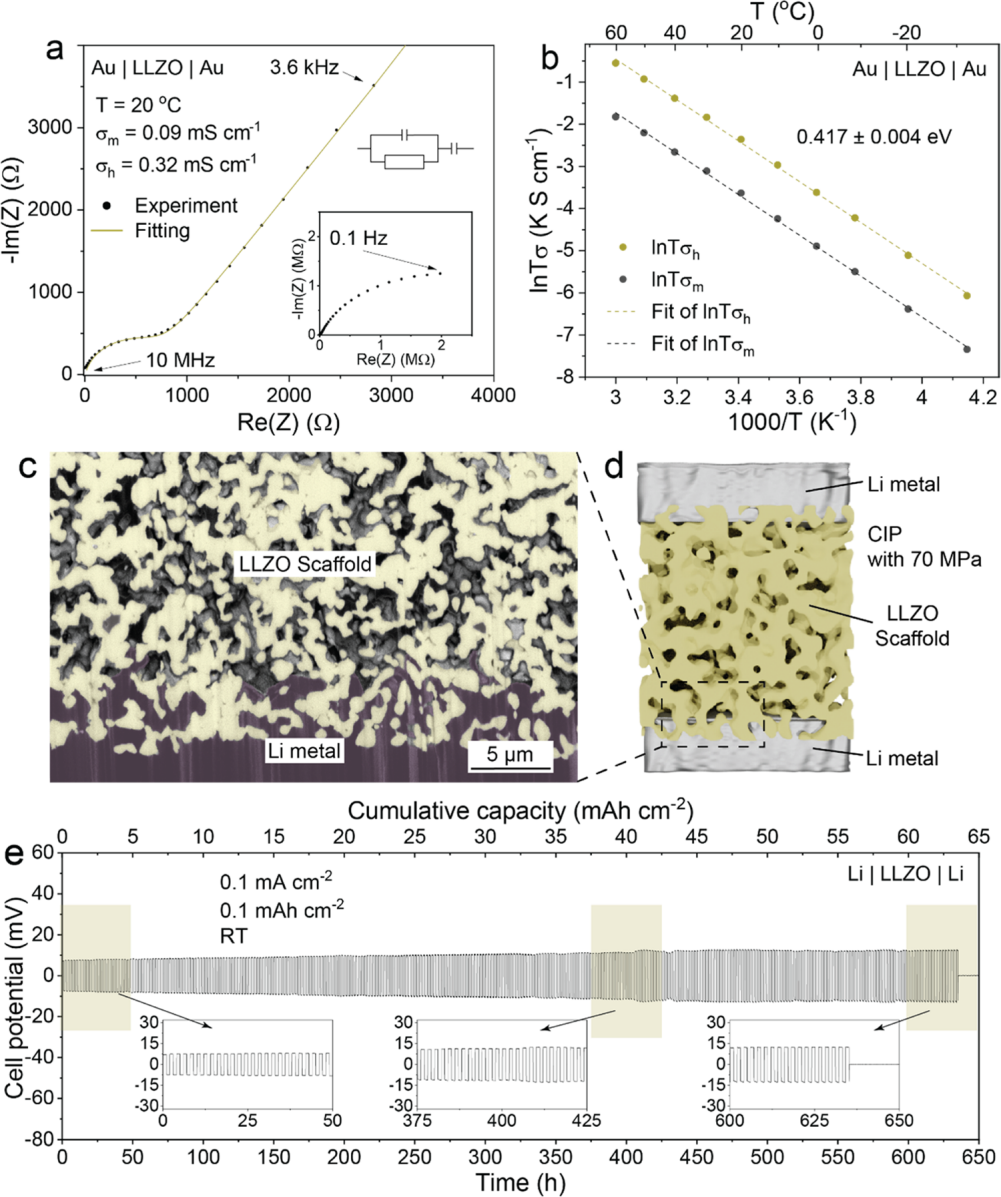
(a) Nyquist diagram of the porous LLZO membrane measured at 20 °C through the Au/porous LLZO/Au symmetrical cell configuration. (b) Arrhenius plots of the measured total conductivity and porosity-corrected total conductivity of the porous LLZO membrane. (c) FIB-SEM image of LLZO membranes infiltrated by Li metal using an isostatic pressure of 70 MPa. (d) Schematics of a Li/porous LLZO/Li symmetrical cell used for electrochemical cycling measurements. (e) Voltage profiles of the Li/LLZO membrane/Li symmetrical cell measured at a current density of 0.1 mA cm^–2^ and with an areal capacity limitation of 0.1 mAh cm^–2^ per half-cycle at room temperature. Refer to Figure S9 for the change in interfacial resistance of the Li/LLZO interface during the cycling of the cell shown in panel (e). Additionally, voltage profiles of the Li/LLZO membrane/Li symmetrical cells measured at higher current densities of 1 and 2 mA cm^–2^, with an areal capacity limitation of 1 mAh cm^–2^, are provided in Figure S10.

The assessment of activation energy and Li-ion conductivity in the prepared LLZO membranes was conducted through temperature-dependent electrochemical impedance spectroscopy (EIS) measurements using a Au/LLZO/Au symmetrical cell configuration (Figures 4a,b and S6). Symmetrical cells were prepared by thermal evaporation of Au electrodes on the surface of LLZO membranes. To consider the impact of porosity on the measured total resistance of the LLZO membranes and therefore to calculate the actual ionic conductivity of the porous LLZO membranes excluding the porosity factor, Bruggeman symmetric medium theory has been used.^43,44^ This theory considers porosity as a low-conductivity phase, linking the measured conductivity (σ_m_) with the membrane’s geometrical dimensions (thickness and electrode area), corrected for porosity (σ_h_), and the volume fraction of porosity (*f*) using the equation . The estimated activation energy and ionic conductivity of the LLZO membranes were approximately 0.417 eV and 0.32 mS cm^–1^, respectively. These values align with those reported in existing literature, affirming consistency.^34^

To explore the electrochemical behavior of self-standing porous LLZO membranes, symmetric Li/LLZO/Li cells were fabricated using the cold isostatic pressing of Li onto an LLZO membrane at ca. 71 MPa. This methodology enabled the impregnation of roughly 5 μm of Li within the 50% porous LLZO scaffold (Figure 4c,d). EIS measurements of the Li/LLZO/Li cells validated a minimal interfacial resistance of 24.6 Ω cm^2^ between metallic Li and the LLZO solid-state electrolyte (Figure S7). For galvanostatic cycling experiments, a capacity limitation of 0.1 mAh cm^–2^ per half-cycle was enforced at a current density of 0.1 mA cm^–2^ without external pressure at room temperature. Figure 4e illustrates the typical galvanostatic voltage profiles of these symmetrical cells. The Li/LLZO/Li cells demonstrated robust cycling stability over a duration of 625 h, equating to ca. 62 mAh cm^–2^ of cumulative Li areal capacity and a consistently stable overpotential of around 10 mV throughout the cycling measurements. It is crucial to note that control experiments conducted using symmetric Li/LLZO/Li cells based on porous LLZO membranes featuring a larger pore size of ca. 5 μm (Figure S4a,d) exhibited considerably lower cyclic stability, lasting only 100 h and achieving a cumulative capacity of ca. 10 mAh cm^–2^ (Figure S8).

**Figure 5:**
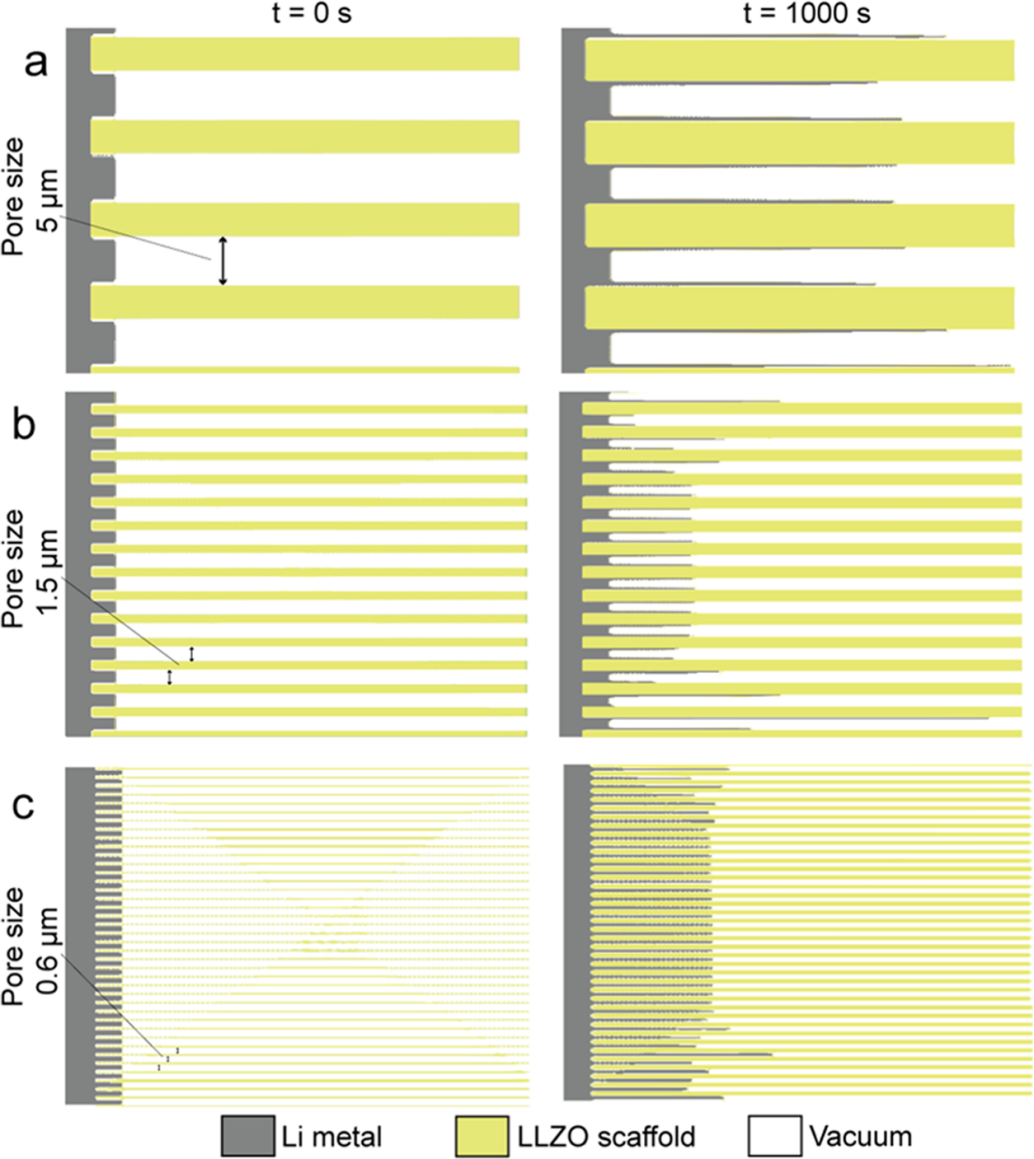
Results of the phase-field simulations of Li growth inside the LLZO scaffolds with three different pore sizes of 5 μm (a), 1.5 μm (b), and 0.6 μm (c). The left figures show the morphologies of Li at *t* = 0 s and the right figures show the morphologies of Li after 1000 s of electrodeposition.

To elucidate the superior electrochemical performance observed in LLZO membranes with smaller pore sizes, we conducted phase-field simulations to investigate the deposition of Li into LLZO scaffolds with varying pore dimensions while maintaining a porosity of 50%. The simulations were performed by using a time-dependent partial differential equations (PDE) solver in COMSOL Multiphysics software, assuming that the LLZO scaffold is composed of vertically aligned channels with a tortuosity of 1. The results of the simulations are shown in Figure 5, which illustrates the growth of the metallic lithium inside of the LLZO scaffolds with different sizes of channels of 5, 1.5, and 0.6 μm, after 1000 s of Li electrodeposition at a current density of 0.2 mA cm^–2^. Prior to electrodeposition of Li, we set the Li/LLZO interface of cells to mimic the as-prepared cells after isostatic pressing, allowing 5 μm of Li impregnation inside the channels. As follows from Figure 5, the electrodeposition of Li within LLZO scaffolds with relatively large pore sizes is rather inhomogeneous, primarily resulting in the formation of metallic lithium along the surface of LLZO channels rather than uniformly filling the entire pores. For LLZO scaffolds with pore sizes of 1.5 μm, a slightly less pronounced inhomogeneous behavior is observed. Conversely, we have identified that LLZO scaffolds with a size of 0.6 μm facilitate homogeneous Li plating, enabling the uniform deposition of metallic lithium inside the pores. These simulation results indicate that reducing the pore size in the LLZO scaffold effectively impedes Li dendrite growth by confining Li deposits within the pores and limiting their expansion along the surface of the LLZO channels.

## Conclusions

In summary, our study presents a novel fabrication method for producing microporous LLZO membranes with a thickness of 50 μm, an average pore size of ca. 2.3 μm, a porosity of ca. 51%, and a specific surface area of 1.3 μm^–1^. The mechanical robustness of the produced LLZO membranes was confirmed by their high breaking force of 146 mN. The core concept behind our approach lies in the utilization of small pore formers combined with the UFS technique, enabling rapid heating and cooling rates of approximately 50 °C per second. This controlled termination of the sintering process prevents the complete densification of the LLZO membranes.

Following the thermal purification of the LLZO membrane surface in both air and argon environments, effectively eliminating carbon and Li_2_CO_3_ impurities, we extensively evaluated the electrochemical properties of the fabricated membranes using a symmetrical Li/LLZO/Li configuration. A thorough comparison between porous LLZO membranes with small and large pore sizes, exhibiting identical porosity of ca. 51%, revealed the superior cycling stability of membranes with smaller pores under identical electrochemical conditions. Specifically, the porous LLZO membranes with average pore sizes of ca. 2.3 μm exhibited high cycling stability, surpassing 600 h at a current density of 0.1 mA cm^–2^, with an areal capacity limit of 0.1 mAh cm^–2^. Conversely, the membranes with pore sizes of ca. 4.7 μm displayed significantly lower cycling stability, not exceeding 100 h. Importantly, the superior cycling stability observed in LLZO membranes with smaller pore sizes was consistent with the results obtained from the phase-field simulations.

## Experimental Section

### Calculations of the Specific Surface Area and Current Density Reduction

To analyze a key property of the porous LLZO structures, the reduction in applied current density resulting from increased Li/LLZO contact area, a simplified model has been employed, assuming that the LLZO scaffold comprises pores in the form of parallel cylinders (see Figure 1b). The distance between these parallel cylinders defines the porosity of the porous structure with a given pore size. Taking into account both the size of the pores and the porosity of the porous LLZO, the specific surface area (*S*_c_) was calculated using the following expression:
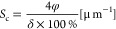
1where φ is the porosity of the scaffold [%] and δ is the pore size (diameter), assuming columns as pores [μm].

The calculation of the current density reduction (*R*_J_), considering the specific surface area and the depth of Li impregnation into the LLZO scaffolds, was performed using the following expression:
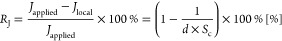
2where *J*_local_ is the local current density [mA cm^–2^], *J*_applied_ is the applied current density [mA cm^–2^], *d* represents the depth of Li impregnation in the LLZO scaffold [μm], and *S*_c_ is the specific surface area of the LLZO scaffold [μm^–1^].

### The Specific Surface Area of LLZO Scaffolds

The specific surface area of LLZO scaffolds was determined using eq 3 considering their pore size and porosity, which were determined by ImageJ from the cross-sectional SEM image shown in Figure 3a (this work) and reported cross-sectional SEM images:
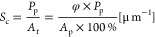
3where φ is the porosity of the scaffold [%], *A_t_* [μm^2^] is the total area of the 2D cross section of the 3D scaffold, and *P*_p_ [μm] and *A*_p_ [μm^2^] are the total pore perimeter and pore area of the 2D cross section, respectively.

### Chemicals

Al-LLZO (Ampcera, 500 nm nanopowder, Al-LLZO), acrylic pore former particles of different diameters (1.5 and 10 μm; Chemisnow MX-150, Soken), a plasticizer (G-260, SEKISUI S-LEC), and a surfactant solution (MALIALIM) were used in this study.

### Preparation of LLZO Slurry and Tape-Casting Process

The LLZO slurry was formulated by blending Al-LLZO powder, acrylic pore former particles ca. 1.5 μm in diameter, surfactant solution, plasticizer, and a solvent mixture comprising 5 vol % isopropanol, 87 vol % ethanol, and 8 vol % 1-propanol. This mixture underwent preliminary mixing using a spatula before undergoing ball-milling at 165 rpm for 18 h. Subsequently, a binder solution [poly(vinyl butyral) in isopropanol] was introduced, and further ball-milling was carried out at 200 rpm for an additional 2 h. The resulting LLZO slurry was tape-cast onto a glass substrate and left for 1 h under ambient conditions for solvent evaporation. It should be noted that the preparation of the slurry for fabricating LLZO scaffolds with larger pore sizes of ca. 10 μm followed a similar procedure, with the only difference being the use of acrylic pore former particles with a diameter of ca. 5 μm. SEM images of both small and large pore former particles, along with their size distribution, can be found in Figure S11. Cross-sectional SEM images of LLZO membranes after each processing step are shown in Figure S12.

### Debinding and Ultrafast Sintering of LLZO Membranes

Initially, the LLZO tapes were sectioned into disks (Ø = 1 cm), placed between two alumina plates, and subjected to a thermal treatment in air at 600 °C. This thermal treatment served multiple purposes: (i) complete removal of the solvent used in the slurry (up to 150 °C), (ii) decomposition of the pore formers (at ca. 350 °C), and (iii) elimination of any residual organic components, including the binder and plasticizer (at ca. 600 °C). Following debinding, the resulting LLZO membranes were positioned between graphite foils, interlaid with carbon plates, and underwent ultrafast sintering at 1150 °C for 20 s in an Ar atmosphere. This sintering process was conducted using a custom-designed setup powered by an AC/DC power source (Aim-TTi CPX400DP Dual 420 W PowerFlex DC Power supply), closely monitoring the temperature with an IR camera (MAURER Pyrometer KTRD 4085-1).^39^ The sintered LLZO membranes were heat-treated at 600 °C for 30 min in an air environment to eliminate any residual graphite. Additionally, a subsequent heat treatment at 900 °C for 10 min in an Ar atmosphere was performed to remove any potential contamination from Li_2_CO_3_ or LiOH on the LLZO surface.

### Material Characterization

The scanning electron microscopy (SEM) images were acquired by using a ZEISS GeminiSEM 460 microscope at an acceleration voltage of 5 kV. Additional SEM images in the Supporting Information were obtained by using a Hitachi TM3030Plus Tabletop microscope, operating at an acceleration voltage of 10 kV.

Cross-sectional FIB-SEM images were obtained using a Thermo Fisher Scientific Helios 5 Multi-Ion-Species Plasma FIB-DualBeam Microscope. Cross sections were prepared using argon as a working gas with an acceleration voltage of 30 kV and a current of up to 2.0 μA. Corresponding SEM images were recorded with a stage tilt of 52°, utilizing an acceleration voltage of 2 kV in secondary electron mode and displayed with tilt correction.

To determine the mechanical strength, a 3-point bending test was conducted on a Tinius Olsen 1ST electromechanical testing machine. The test involved applying a crosshead speed (s) of 1 μm s^–1^ and a strain rate (ε) of 1.56 × 10^–5^ s^–1^ to the sample. Flexural strength (σ_f_) and strain rate (ε) were calculated using the following formulas:
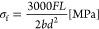
4and
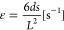
5where *b* is the width of the LLZO membrane (5.0 mm, in the shape of a square), *d* is the thickness of the LLZO membrane (65 μm), *F* is the breaking force (mN), and *L* is the support span (5 mm).

Raman spectroscopy involved using a confocal Raman microscope (Horiba, LabRAM HR Evolution) with a 532 nm Nd:Yag laser (Cobolt SambaTM). To avoid exposure to air, LLZO samples were sealed between thin glass slides by using epoxy glue inside an Ar-filled glovebox.

X-ray computed tomography measurements were conducted using an RX Solutions Easy Tom XL system with a voxel size of 850 nm. Image reconstruction was performed using X-Act computed tomography software (RX Solutions, Chavanod, France). Quantitative 3D and 2D analyses of reconstructed images were performed using GeoDict software.

X-ray photoelectron spectroscopy (XPS) analysis was carried out using a PHI Quantes spectrometer (ULVAC-PHI) equipped with a low-energy Al-Kα source (1486.6 eV). The XPS spectrometer was directly linked to an Ar-filled glovebox, allowing in situ transfer of LLZO samples without exposure to air.

The level of activation energy of LLZO membranes was evaluated through temperature-dependent electrochemical impedance spectroscopy (EIS) measurements, employing a Au/LLZO/Au cell configuration. The Au electrodes were deposited onto LLZO membranes by using a Covap thermal evaporator (Angstrom). Ionic conductivities (σ_m_) were calculated from the total resistance value of porous LLZO membranes (*R*_total_), factoring in their thickness (65 μm) and the electrode surface area (0.1257 cm^2^), using the formula:
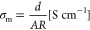
6where *d* is the thickness of the membrane [cm], *A* is the area of the electrode [cm^2^], and *R* is the resistance [Ohm].

The actual ionic conductivity of porous LLZO membranes, considering their porosity, was calculated using the Bruggeman symmetric medium relationship:^43,44^
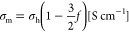
7where σ_h_ represents the porosity-corrected conductivity and *f* is the volume fraction of porosity.

### Preparation of Symmetrical Cells

Symmetric Li/LLZO scaffold/Li cells were fabricated through cold isostatic pressing of Li foil onto both sides of the porous LLZO membrane at a pressure of ca. 71 MPa for 5 min. Electrochemical impedance spectroscopy (EIS) measurements were then performed in the frequency range of 1 MHz to 10 Hz, using a Li/LLZO scaffold/Li cell configuration, with an amplitude of 10 mV.

### Phase-Field Simulations

To investigate the impact of the LLZO pore size on Li plating, we developed a two-dimensional phase-field model. The model assumes that the LLZO scaffold comprises vertically aligned channels with a tortuosity of 1. It employs two order parameters, denoted as ξ and ϕ, representing the lithium anode and the LLZO membrane, respectively. These parameters continuously transition from 0 to 1 at the interface, signifying ξ = 1 in the Li anode and ϕ = 1 in the LLZO membranes. The system’s total free energy is formulated by
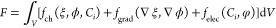
8where *f*_grad_ is a gradient energy density, *f*_ch_ is the Helmholtz free energy density, and *f*_elec_ is the electrostatic energy density. The Helmholtz free energy density is the combination of the energy of ion mixing (*f*_ion_) and the local free energy density (*f*_0_). The local free energy can be expressed as , where *W*_1_ and *W*_2_ are the energy barrier height (J m^–3^) of the double well function for order parameters ξ and ϕ, respectively, and  is a cross term added to the free energy function to yield three equilibrium conditions: for lithium electrode (ξ = 1, ϕ = 0), for LLZO membranes (ξ = 0, ϕ = 1), and pores (ξ = 0, ϕ = 0). Similarly, the gradient energy density is written as , where κ_1_ and κ_2_ are the gradient energy coefficients (J m^–1^) of the lithium anode and LLZO membranes, respectively. Dendrite growth is simulated by adding an anisotropic gradient energy coefficient for order parameter ξ, i.e., κ_1_ = κ_0_ [1 + δ cos(*w*θ)], where κ_0_ is related to the surface energy of Li, δ is the strength of anisotropy, *w* is the mode of anisotropy, and θ is the angle between the reference axis and normal vector to the interface. Lastly, the electrostatic energy takes the form *f*_elec_ (*c*_*i*_,φ) = *F*∑_*i*_*z*_*i*_*c*_*i*_φ, where *F* is the Faraday constant (C mol^–1^), φ is the electric potential (V), *z*_*i*_ is the charge number, and *c*_*i*_ is the concentration (mol m^–3^) of the charged species. More detailed descriptions of energy densities can be found in ref (45).

The deposition of the Li^+^ anode during the cycling process is expressed by the temporal evolution of order parameter ξ. Taking into account the Butler–Volmer kinetics, the growth rate of the Li anode can be expressed by the following equation:
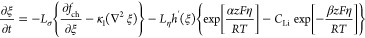
9where *L*_σ_ is the interface mobility (m^3^ J^–1^ s^–1^), *L*_η_ is the reaction rate (s^–1^), η is the over-potential (V), *z* is the charge number, *R* is the gas constant (J mol^–1^ K^–1^), α and β are the symmetric factors, *h*′(ξ) is the first derivative of the interpolation function *h*(ξ), and *T* is the absolute temperature (K).

The order parameter ϕ is assumed to be nonevolving; hence, its equation can be written as follows:
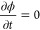
10The evolution of Li^+^ concentration can be expressed by the Nernst–Planck equation as follows:
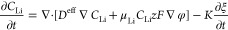
11where *D*^eff^ is the effective diffusivity (m^2^ s^–1^) of Li^+^, μ_Li_ is the mobility (m^2^ V^–1^ s^–1^) of Li^+^, and K is the annihilation/accumulation rate (mol L^–1^) of Li^+^ due to reaction at the Li surface (Li^+^ + e^–^ → Li). The effective diffusivity takes the form *D*^eff^ = *D*_e_*h*(ξ) + *D*_p_*h*(ϕ) + *D*_s_(1 – *h*(ξ) – *h*(ϕ)), where *D*_e_, *D*_p_, and *D*_s_ are the diffusivity of Li^+^ in the electrode, LLZO scaffolds, and pores, respectively; *h*(ξ) = ξ^3^ (6ξ^2^ – 15ξ + 10) and *h*(ϕ) = ϕ^3^ (6ϕ^2^ – 15ϕ + 10) are two interpolation functions. We assumed that LLZO pores are vacuum; hence, *D*_s_ = 0.

The half-cell battery system is assumed to be charge neutral. The potential distribution in the system is calculated by solving the current continuity equation,
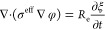
12where σ^eff^ is the effective electrical conductivity (S m^–1^) and *R*_e_ is the current constant. σ^eff^ is calculated by using interpolation functions, i.e., σ^eff^ = σ^e^*h*(ξ) + σ^p^*h*(ϕ) + σ^s^(1 – *h*(ξ) – *h*(ϕ)), where σ^e^, σ^p^, and σ^s^ are the electrical conductivities (S m^–1^) of the Li anode, LLZO scaffolds, and pores, respectively. Since the pores are in a vacuum, σ^s^ = 0.

Equations 9–12 were solved together using a time-dependent PDE solver in COMSOL Multiphysics software. The simulation settings included a system size of 50 × 50 μm^2^, manually applied mesh size, and a maximum elemental size of 0.25 μm. Dirichlet boundary conditions were imposed for variables *C*_Li_ and φ, while no flux boundary conditions were set for order parameters ξ and ϕ. The simulations were performed at room temperature (*T* = 298 K).

## Supporting Information Available

Additional experimental details, supporting Figures S1–S12, Tables S1 and S2 (PDF)
