# Supplementary material for: Garnet-Based Solid-State Li Batteries with High-Surface-Area Porous LLZO Membranes
Source: ACS Appl Mater Interfaces. 2024 Mar 4;16(10):12353–62. doi: 10.1021/acsami.3c14422 (PMC10941065; doi:10.1021/acsami.3c14422)
Supplement: Supplementary file 1 — am3c14422_si_001.pdf [file am3c14422_si_001.pdf]

Supporting Information for

Garnet-Based Solid-State Li Batteries with High-Surface-Area Porous LLZO Membranes

Huanyu Zhang, [1,2] *Faruk Okur,*[1,2] *Bharat Pant,*[3] *Matthias Klimpel,*[1,2] *Sofiia Butenko,* [1,2] *Dogan Tarik Karabay,*[1,2]*Annapaola Parrilli,*[4] *Antonia Neels,*[4] *Ye Cao,*[3] *Kostiantyn V. Kravchyk,*[1,2*] *Maksym V. Kovalenko*[1,2*]

[1] Laboratory for Thin Films and Photovoltaics, Empa – Swiss Federal Laboratories for Materials Science and Technology, Überlandstrasse 129, CH-8600 Dübendorf, Switzerland

[2] Laboratory of Inorganic Chemistry, Department of Chemistry and Applied Biosciences, ETH Zürich, Vladimir-Prelog-Weg 1, CH-8093 Zürich, Switzerland

[3]Department of Materials Science and Engineering, The University of Texas at Arlington, Arlington, TX, 76019, USA

[4]Center for X-ray Analytics, Empa - Swiss Federal Laboratories for Materials Science & Technology, CH-8600 Dübendorf, Switzerland

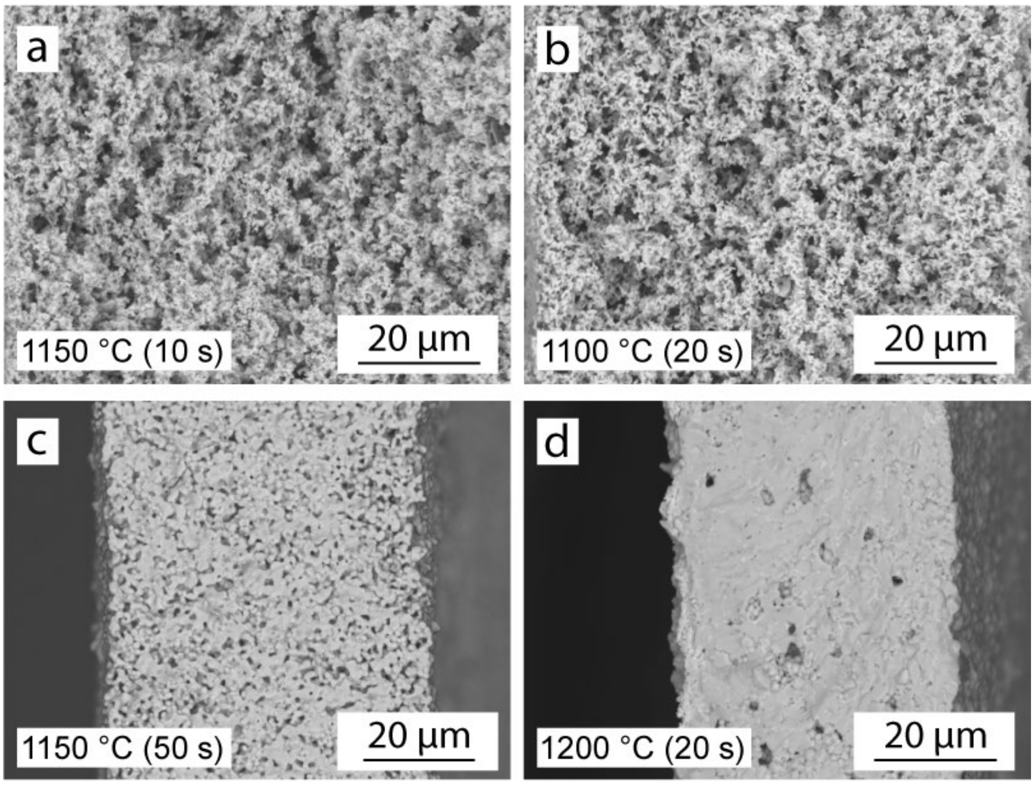

Figure S1. Cross-section SEM images of LLZO membranes sintered at different sintering temperatures and times: 1150 °C, 10 sec (**a**), 1100 °C, 20 sec (**b**), 1150 °C, 50 sec (**c**) and 1200 °C, 20 sec (**d**).

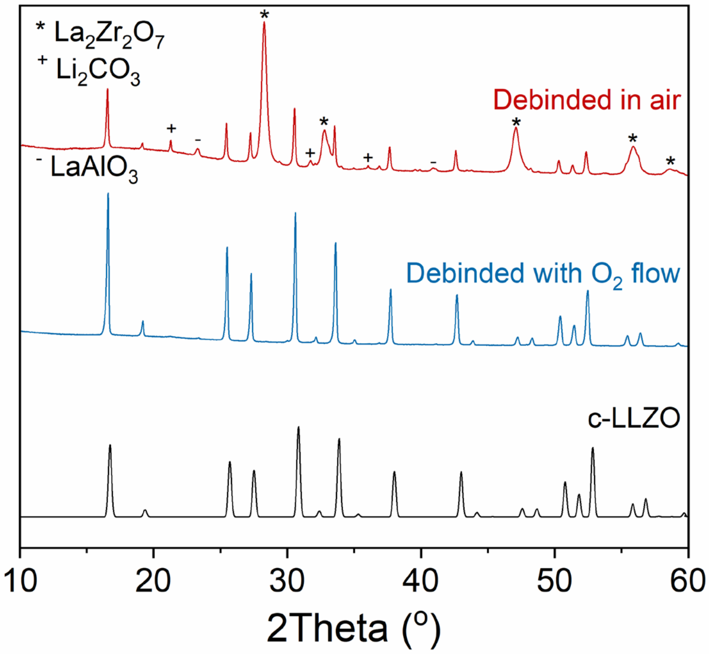

Figure S2. Comparison of X-ray diffraction patterns of LLZO tapes after de-binding at 600 °C under O_2 flow and in air (without flow). X-ray diffraction pattern of c-LLZO (ICSD:235896) is given for comparison.

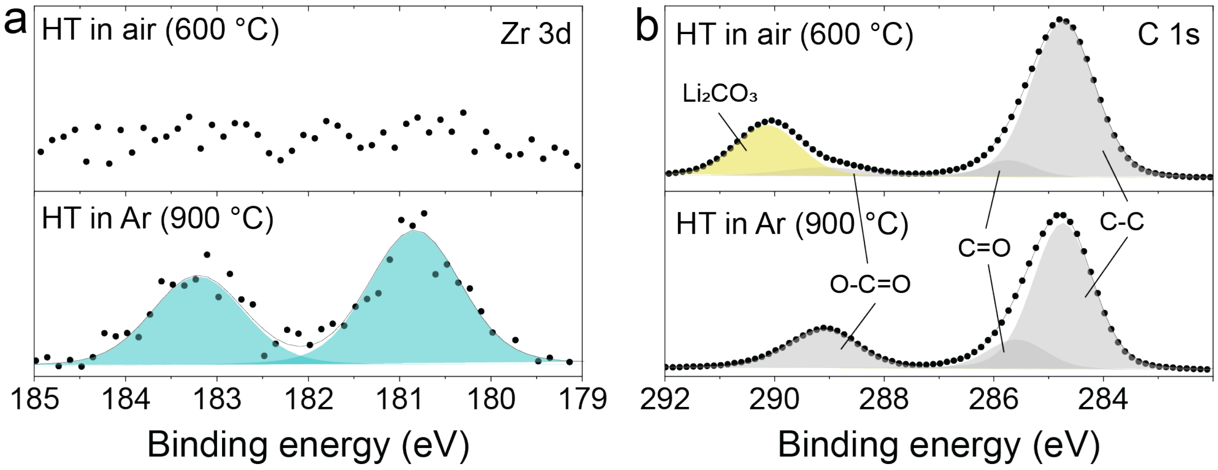

Figure S3. Charge-corrected Zr 3d (a) and O 1s (b) spectra of ultrafast-sintered LLZO membranes after heat-treatment in air and a followed heat-treatment step in an Ar-filled glovebox at 900 °C. The absence of the Zr 3d peaks and the concurrent presence of C 1s peaks associated with both the LLZO structure and Li_2CO_3 indicate substantial surface contamination by Li_2CO_3 on ultrafast-sintered LLZO membranes after heat treatment in air. Conversely, the XPS data for LLZO membranes subjected to a subsequent heat-treatment step in an Ar-filled glovebox at 900 °C present a contrasting scenario, demonstrating the presence of Zr 3d peaks and the absence of C 1s peaks associated with Li_2CO_3, thus confirming the successful elimination of Li_2CO_3 after Ar heat-treatment.

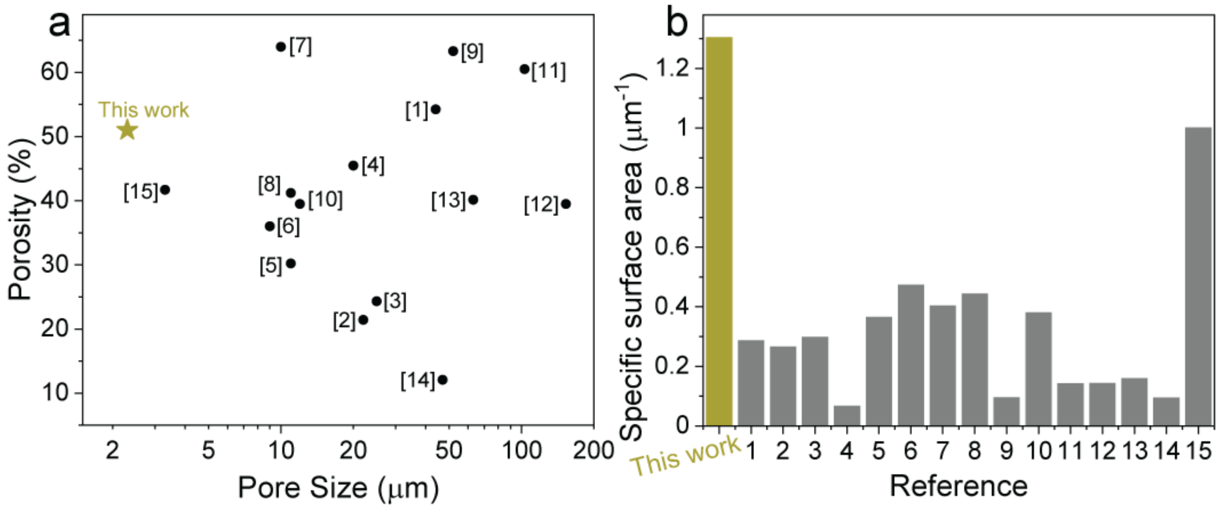

Figure S4. Comparison of pore size and porosity (**a**) and specific surface area (**b**) of fabricated porous LLZO membranes (this work) with reported LLZO scaffolds.[1-15]

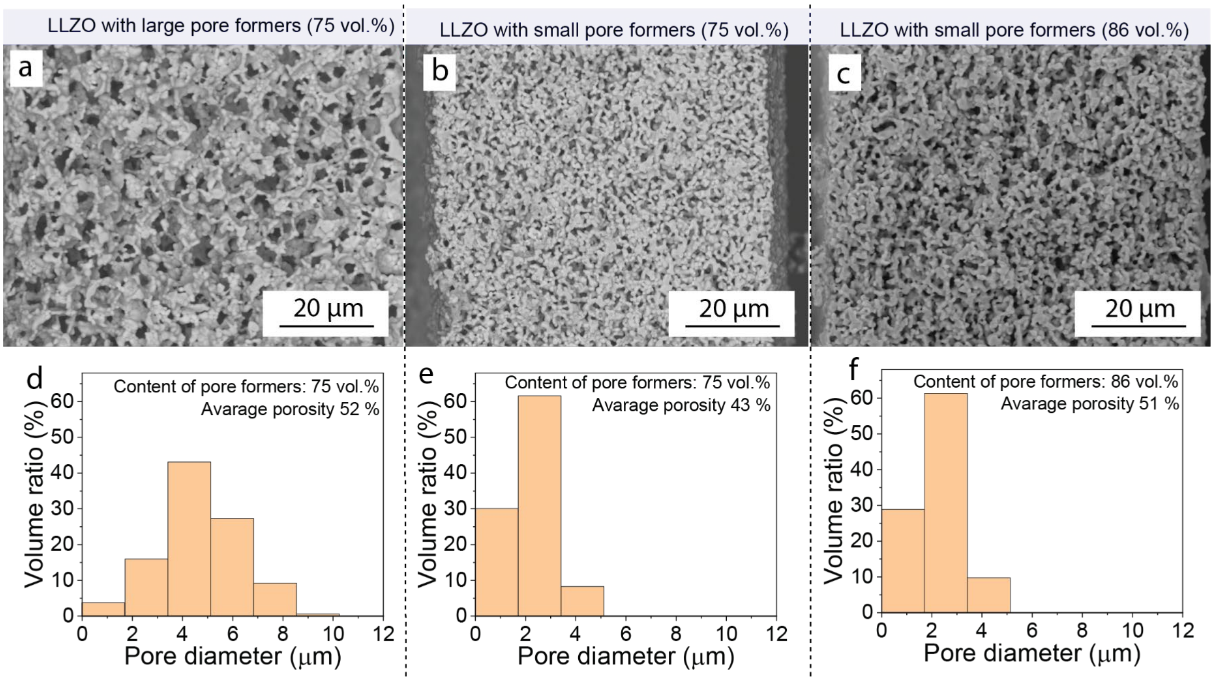

Figure S5. Cross-section SEM images (**a-c**) and corresponding pore size histograms (**d-f**) of porous LLZO membranes prepared using large [10 µm (75 vol.%)] and small (1.5 µm (75 vol.% and 86 vol.%)] pore formers.

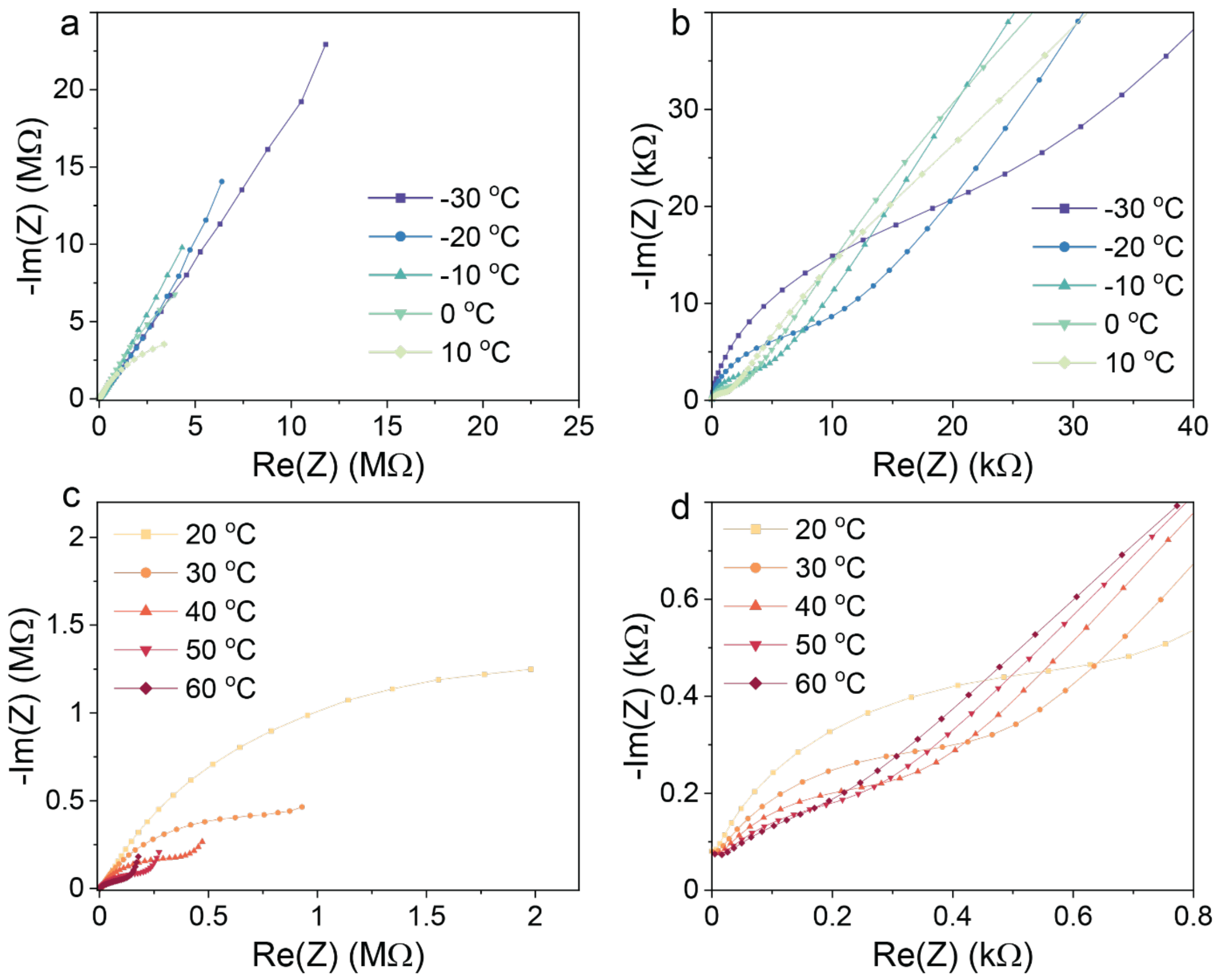

Figure S6. Impedance spectra of porous LLZO membrane with small pores (2.3 μm) measured in the temperature range of -30 °C to 10 °C (**a**, **b**) and 20°C to 60 °C (**c**, **d**) using Au/LLZO/Au configuration.

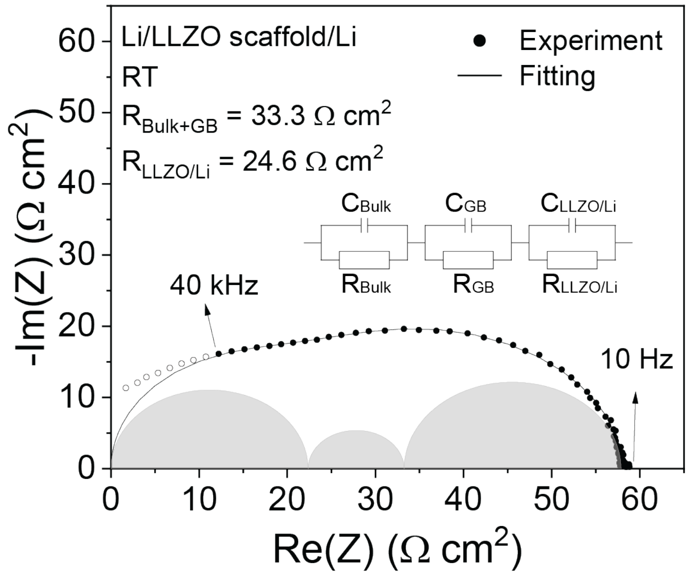

Figure S7. Impedance spectra of Li/LLZO/Li symmetrical cell based porous LLZO membranes with small pores of 2.3 μm, measured at room temperature.

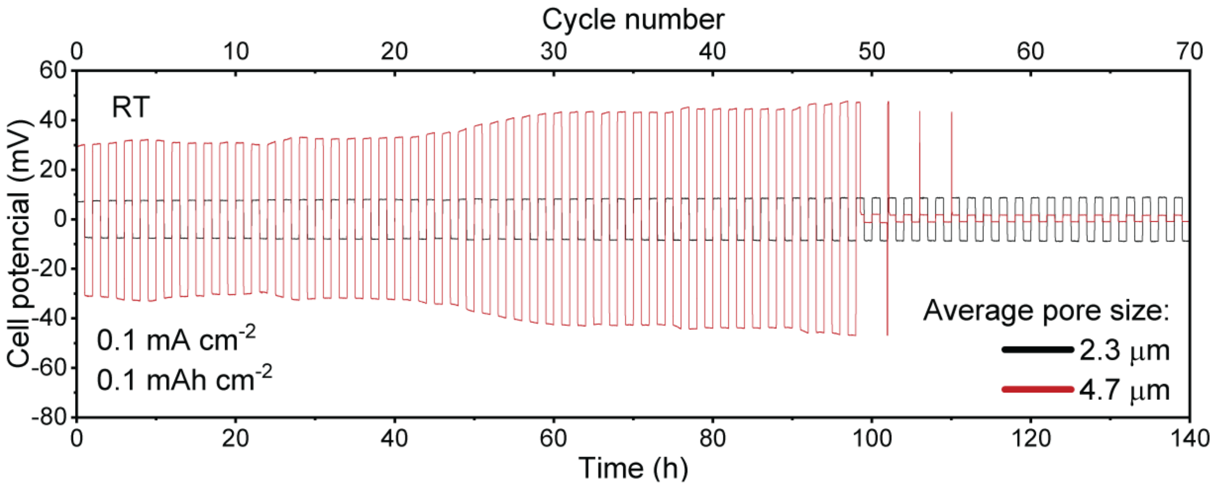

Figure S8. Voltage profiles of the Li/LLZO/Li symmetrical cell based on LLZO membranes with small (2.3 μm) and large (4.7 μm) pores measured at a current density of 0.1 mA cm^{-2} and an areal capacity limitation of 0.1 mAh cm^{-2} per half-cycle at room temperature.

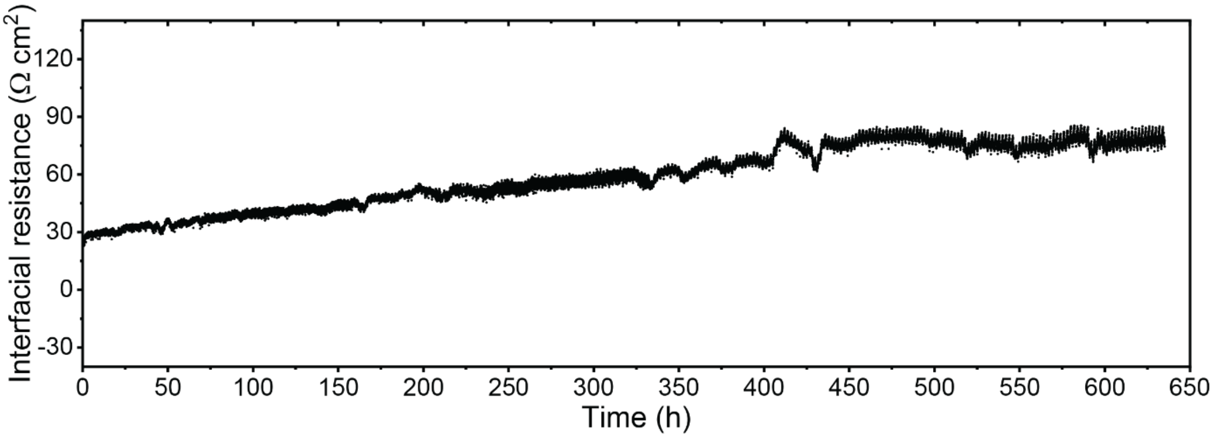

Figure S9. The change of the interfacial resistance of Li/LLZO interface during galvanostatic cycling at a current density of 0.1 mA cm^{-2} and the areal capacity limitation of 0.1 mAh cm^{-2}. The interfacial resistance at a given cycle number was calculated by summing the initial interfacial resistance value (determined from the EIS spectra of the as-prepared Li/LLZO/Li symmetrical cells prior to cycling, see **Figure S7**) and the increase in the interfacial resistance of the cell at a given cycle compared to the initial cycle. The interfacial resistance of the cell at a given cycle was calculated by applying Ohm's law using the current density of 0.1 mA cm^{-2} and the observed overpotential at the given cycle number. Voltage profiles of the Li/LLZO membrane/Li symmetrical cell cycled at a current density of 0.1 mA cm^{-2} and an areal capacity limitation of 0.1 mAh cm^{-2} per half-cycle are shown in **Figure 4e**.

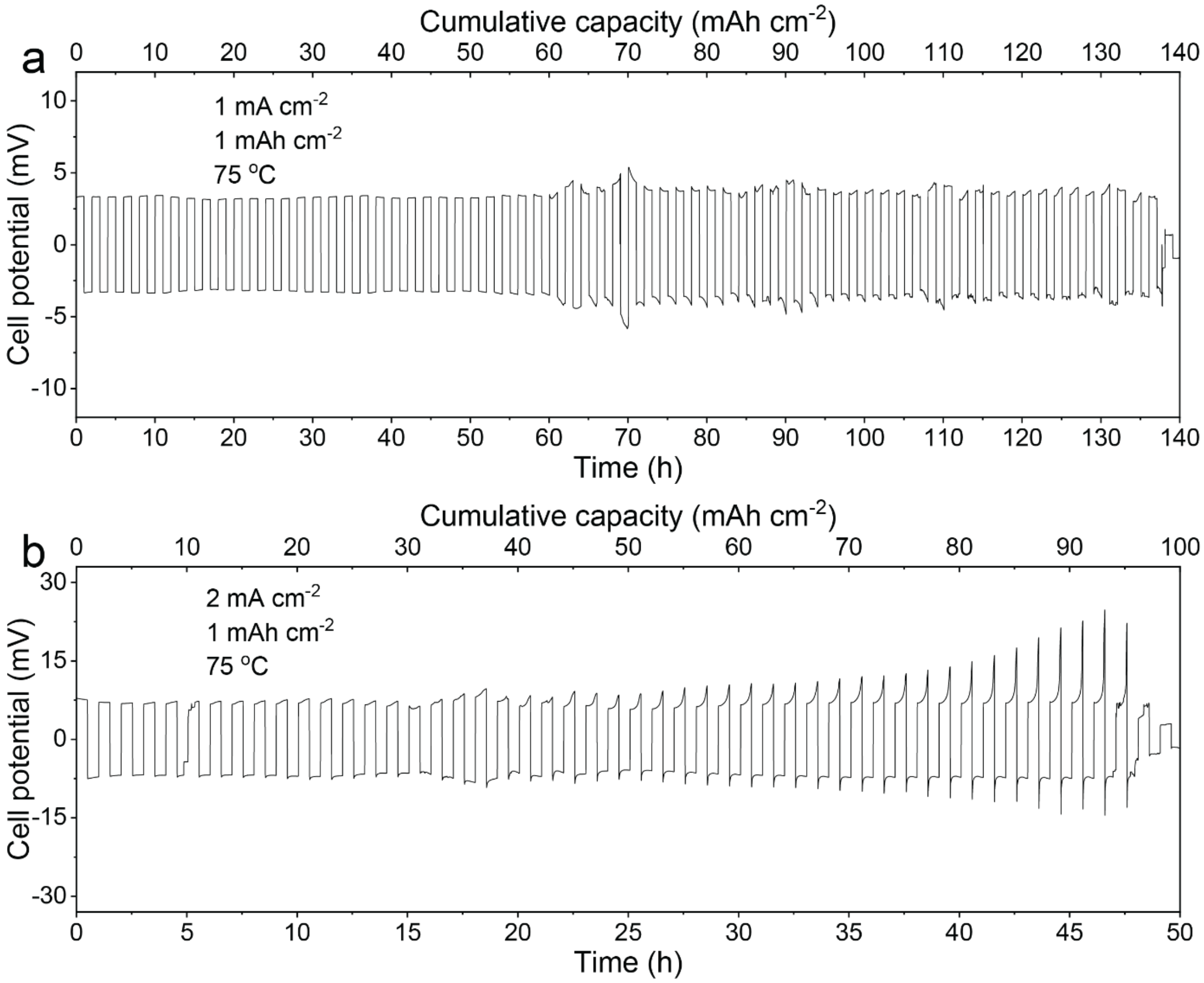

Figure S10. Voltage profiles of the Li/LLZO membrane/Li symmetrical cell measured at a current densities of 1 mA cm^{-2} and 2 mA cm^{-2} and an areal capacity limitation of 1 mAh cm^{-2} per half-cycle at 75 °C.

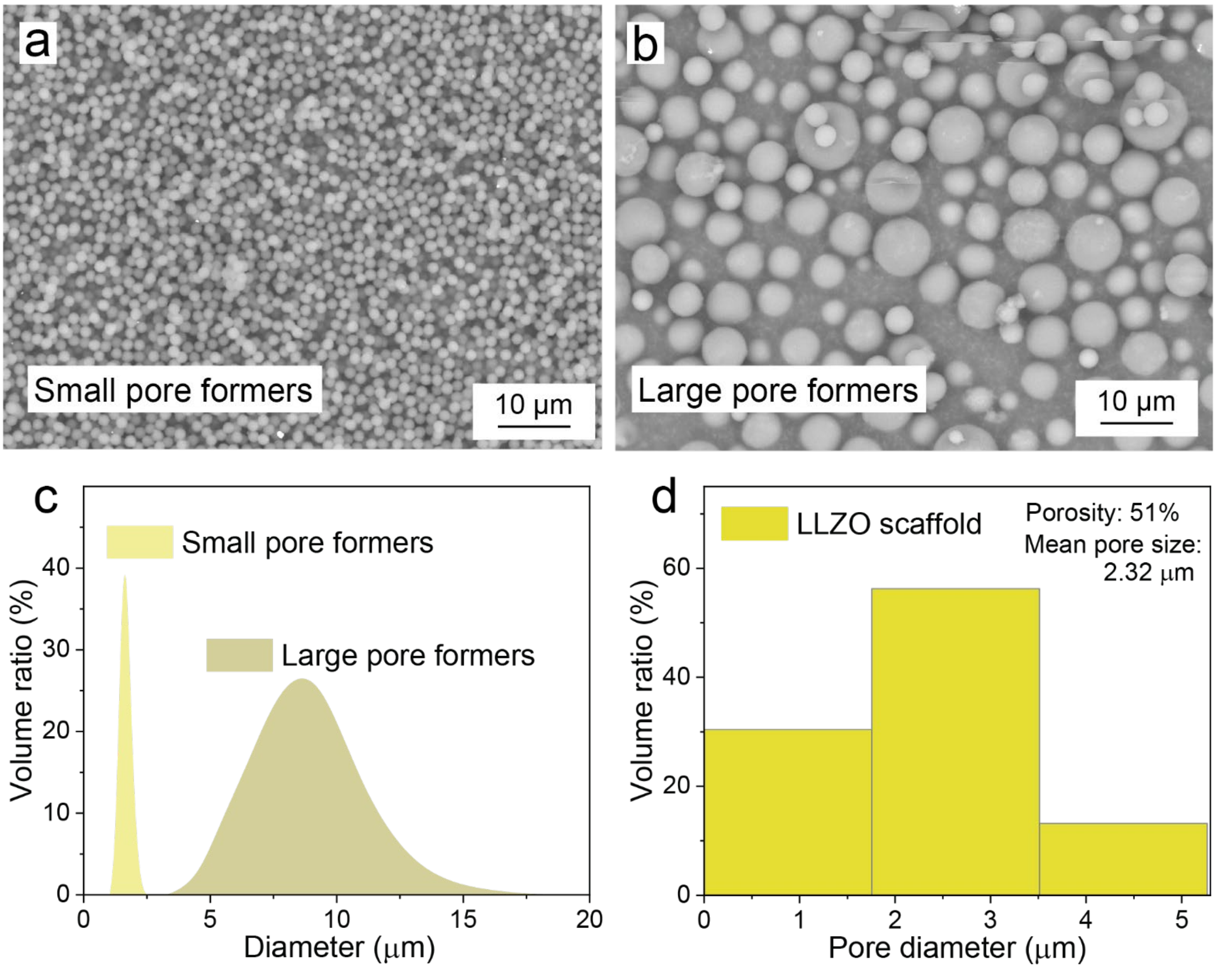

Figure S11. SEM images of small (**a**) and large (**b**) pore formers along with their size histograms (**c**). (**d**) Size histogram of the pores inside the LLZO membrane.

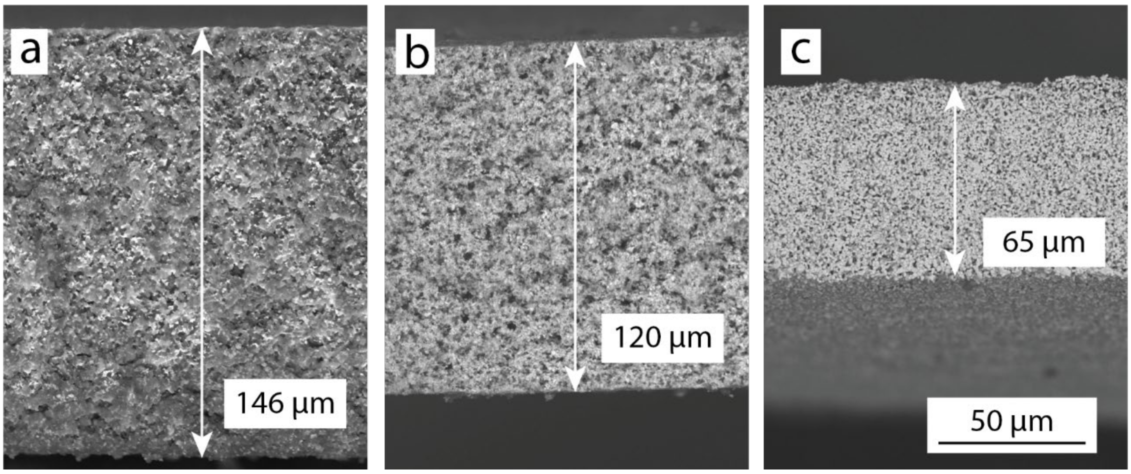

Figure S12. Cross-sectional SEM images of LLZO membranes at different processing stages: after (**a**) tape-casting and drying, (**b**) de-binding and (**c**) sintering.

Table S1. Comparison of pore size, porosity and specific surface area of fabricated porous LLZO membranes (this work) with reported LLZO scaffolds. The specific surface area of LLZO scaffolds was calculated using equation (3) considering their pore size and porosity, which were determined by ImageJ from cross-sectional SEM image shown in Figure 3a (this work) and reported cross-sectional SEM images (references [1]-[15]).

Ref	A_t (μm^2)	A_p (μm^2)	P_p (μm)	φ (%)	δ_{mean} (μm^2)	S_c (μm^{-1})
**This work**	**1939**	**930.039**	**2529.164**	**48.0**	**2.4**	**1.304**
[1]	32400	17574.1	9276.82	54.2	44	0.286
[2]	1642	351.839	436.478	21.4	22	0.266
[3]	14760	3590.13	4397.11	24.3	25	0.298
[4]	1167160	530679	77595.2	45.5	20	0.0665
[5]	8347	2522.09	3050.58	30.2	11	0.365
[6]	5465	1967.70	2586.93	36.0	9.0	0.473
[7]	91139	33911.5	36782.7	64.0	10	0.404
[8]	3839	1582.31	1701.34	41.2	11	0.443
[9]	64562	40881.0	6175.75	63.3	52	0.0957
[10]	16886	6162.89	6423.71	39.5	12	0.380
[11]	84299	27322.8	11938.1	60.5	103	0.142
[12]	44887	14601.0	6416.95	39.5	153	0.143
[13]	128254	51496.1	20481.3	40.2	63	0.160
[14]	63643	7690.70	5988.67	12.1	47	0.0941
[15]	2565	1069.43	2583.55	41.7	3.3	1.001

Table S2. Porosity analysis of sintered LLZO membrane with small pores of *ca*. 2.3 μm. The analysis was conducted using GeoDict software, based on X-ray computed tomography data.

Parameter	Value
**Sample size (μm)**	383 × 328 × 39
**Overall Porosity (%)**	51.3753
**Open Porosity (%)**	51.152
**Closed Porosity (%)**	0.2233

Parameter	Value
**Number of Pores**	12596
**Number of Open Pores**	1560
**Number of Closed Pores**	11036

References

(1) van den Broek, J.; Afyon, S.; Rupp, J. L. M. Interface-Engineered All-Solid-State Li-Ion Batteries Based on Garnet-Type Fast Li+ Conductors. *Adv. Energy Mater.* **2016**, *6* (19).

(2) Ren, Y. Y.; Liu, T.; Shen, Y.; Lin, Y. H.; Nan, C. W. Garnet-type oxide electrolyte with novel porous-dense bilayer configuration for rechargeable all-solid-state lithium batteries. *Ionics* **2017**, *23* (9), 2521-2527.

(3) Fu, K.; Gong, Y. H.; Hitz, G. T.; McOwen, D. W.; Li, Y. J.; Xu, S. M.; Wen, Y.; Zhang, L.; Wang, C. W.; Pastel, G.; et al. Three-dimensional bilayer garnet solid electrolyte based high energy density lithium metal-sulfur batteries. *Energy Environ. Sci.* **2017**, *10* (7), 1568-1575.

(4) Buannic, L.; Naviroj, M.; Miller, S. M.; Zagorski, J.; Faber, K. T.; Llordes, A. Dense freeze-cast Li7La3Zr2O12 solid electrolytes with oriented open porosity and contiguous ceramic scaffold. *J. Am. Ceram. Soc.* **2019**, *102* (3), 1021-1029.

(5) Xu, S. M.; McOwen, D. W.; Zhang, L.; Hitz, G. T.; Wang, C. W.; Ma, Z. H.; Chen, C. J.; Luo, W.; Dai, J. Q.; Kuang, Y. D.; et al. All-in-one lithium-sulfur battery enabled by a porous-dense-porous garnet architecture. *Energy Storage Mater.* **2018**, *15*, 458-464.

(6) Liu, B. Y.; Zhang, L.; Xu, S. M.; McOwen, D. W.; Gong, Y. H.; Yang, C. P.; Pastel, G. R.; Xie, H.; Fu, K.; Dai, J. Q.; et al. 3D lithium metal anodes hosted in asymmetric garnet frameworks toward high energy density batteries. *Energy Storage Mater.* **2018**, *14*, 376-382.

(7) Xu, S. M.; McOwen, D. W.; Wang, C. W.; Zhang, L.; Luo, W.; Chen, C. J.; Li, Y. J.; Gong, Y. H.; Dai, J. Q.; Kuang, Y. D.; et al. Three-Dimensional, Solid-State Mixed Electron-Ion Conductive Framework for Lithium Metal Anode. *Nano Lett.* **2018**, *18* (6), 3926-3933.

(8) Yang, C. P.; Zhang, L.; Liu, B. Y.; Xu, S. M.; Hamann, T.; McOwen, D.; Dai, J. Q.; Luo, W.; Gong, Y. H.; Wachsman, E. D.; et al. Continuous plating/stripping behavior of solid-state lithium metal anode in a 3D ion-conductive framework. *P. Natl. Acad. Sci.* **2018**, *115* (15), 3770-3775.

(9) Shen, H.; Yi, E. Y.; Amores, M.; Cheng, L.; Tamura, N.; Parkinson, D. Y.; Chen, G. Y.; Chen, K.; Doeff, M. Oriented porous LLZO 3D structures obtained by freeze casting for battery applications. *J Mater. Chem. A* **2019**, *7* (36), 20861-20870.

(10) Hitz, G. T.; McOwen, D. W.; Zhang, L.; Ma, Z. H.; Fu, Z. Z.; Wen, Y.; Gong, Y. H.; Dai, J. Q.; Hamann, T. R.; Hu, L. B.; et al. High-rate lithium cycling in a scalable trilayer Li-garnet-electrolyte architecture. *Mater. Today* **2019**, *22*, 50-57.

(11) Yi, E.; Shen, H.; Heywood, S.; Alvarado, J.; Parkinson, D. Y.; Chen, G. Y.; Sofie, S. W.; Doeff, M. M. All-Solid-State Batteries Using Rationally Designed Garnet Electrolyte Frameworks. *ACS Appl. Energ. Mater.* **2020**, *3* (1), 170-175.

(12) Zhao, C. T.; Sun, Q.; Luo, J.; Liang, J. N.; Liu, Y. L.; Zhang, L.; Wang, J. W.; Deng, S. X.; Lin, X. T.; Yang, X. F.; et al. 3D Porous Garnet/Gel Polymer Hybrid Electrolyte for Safe Solid-State Li-O-2 Batteries with Long Lifetimes. *Chem. Mater.* **2020**, *32* (23), 10113-10119.

(13) Kim, K. J.; Rupp, J. L. M. All ceramic cathode composite design and manufacturing towards low interfacial resistance for garnet-based solid-state lithium batteries. *Energy Environ. Sci.* **2020**, *13* (12), 4930-4945.

(14) Shen, F. Y.; Jonson, R. A.; Parkinson, D. Y.; Tucker, M. C. Preparing Li-garnet electrodes with engineered structures by phase inversion and high shear compaction processes. *J. Am. Ceram. Soc.* **2022**, *105* (1), 90-98.

(15) Okur, F.; Zhang, H. Y.; Karabay, D. T.; Muench, K.; Parrilli, A.; Neels, A.; Dachraoui, W.; Rossell, M. D.; Cancellieri, C.; Jeurgens, L. P. H.; et al. Intermediate-Stage Sintered LLZO Scaffolds for Li-Garnet Solid-State Batteries. *Adv. Energy Mater.* **2023**, *13* (15), 2203509.
